# Editorial: Translational Research for Cucurbit Molecular Breeding: Traits, Markers, and Genes

**DOI:** 10.3389/fpls.2020.615346

**Published:** 2020-11-30

**Authors:** Yiqun Weng, Jordi Garcia-Mas, Amnon Levi, Feishi Luan

**Affiliations:** ^1^United States Department of Agriculture, Agriculture Research Service, Vegetable Crops Research Unit, Horticulture Department, University of Wisconsin, Madison, WI, United States; ^2^Center for Research in Agricultural Genomics (CRAG) Consejo Superior de Investigaciones Científicas - Institut de Recerca i Tecnologia Agroalimentàries - Universitat Autònoma de Barcelona - Universitat de Barcelona, Barcelona, Spain; ^3^Genomics and Biotecnology Program, Institut de Recerca i Tecnologia Agroalimentáries (IRTA), Barcelona, Spain; ^4^US Vegetable Laboratory, United States Department of Agriculture, Agriculture Research Service, Charleston, SC, United States; ^5^Key Laboratory of Biology and Genetic Improvement of Horticulture Crops (Northeast Region), Ministry of Agriculture and Rural Affairs, Northeast Agricultural University, Harbin, China

**Keywords:** cucurbits, molecular breeding, QTL mapping, translational genomics, marker-assisted selection

## Introduction

Cucurbits (family Cucurbitaceae) are economically important vegetable crops. Major cucurbits growing globally include cucumber, melon, watermelon, and squash/pumpkin. Other cucurbits like bitter melon, bottle gourd, winter melon, and luffa are popular in many Asian and African countries. The last decade has witnessed a rapid development of genetic and genomics resources including draft genome assemblies, and high-density genetic maps in a dozen cucurbit crops, making it possible to accelerate translational research for cucurbit breeding. This Research Topic is a collection of 21 Original Research articles or Reviews highlighting the achievements and future directions in cucurbit translational research. These articles cover a variety of topics ranging from improvement of the cucurbit genome assemblies to identification and molecular mapping of horticulturally important genes or QTL for horticultural traits, and the use of such knowledge in marker-assisted selection for cucurbit improvement. Major findings from these investigations are summarized below.

## Improvement of Melon Draft Genome Assembly

Draft genomes of nearly a dozen cucurbit crops are now available (https://www.cucurbitgenomics.org/) and are constantly being revised using new technologies and experimental data. Castanera et al. reported a further improvement of the melon reference genome using PacBio single-molecule real time (SMRT) sequencing, which produced melon genome version v4.0 (DHL92). The melon reference genome v3.5.1 (Garcia-Mas et al., 2012) was obtained using 454 technology and the genome assembly was further improved using optical mapping to produce v3.6.1 (Ruggieri et al., 2018). However, v3.6.1 still contained 19% of gaps and more than 40 Mb unassigned sequences, probably missing complex repeat regions. DHL92 melon assembly v4.0 had an increase of the melon genome pseudomolecule size by 40 Mb with 90% of the v3.5.1 gaps being filled, and the completeness was improved mainly in non-genic regions. Specifically, 40% more full-length LTR retrotransposons were identified in the new assembly, mainly located in centromeric and pericentromeric regions, and a burst of these repetitive elements was found to occur less than two million years ago. Some of these elements are polymorphic among melon varieties and sit in the upstream regions of genes; however, their potential regulatory roles are unknown.

## QTL Mapping of Disease Resistances in Cucurbits

Cucurbits are susceptible to a number of common pathogens. Identification of disease resistances and associated molecular markers is often a priority in most cucurbit breeding programs. Three articles reported molecular mapping of host resistances against viral pathogens. Pascual et al. describe a general strategy for virus resistance in melon, conferred by the *vacuolar protein sorting 41* (*CmVPS41*) gene, which is responsible for the restriction of the virus to the bundle sheath cells and preventing phloem entry. VPS41 is a protein involved in intracellular trafficking of proteins and vesicles from late Golgi to the vacuole. A previous study (Giner et al., 2017) found that the *cmv1* locus in melon accession PI 161375 for resistance against the Cucumber Mosaic Virus (CMV) was due to a mutation in *CmVPS41*. Pascual et al. sequenced the *CmVPS41* locus in 52 melon accessions and identified 16 new haplotypes, 12 protein variants, and nine new resistant accessions. Only two non-synonymous mutations, L348R carried by PI 161375 and G85E, resulted in CMV resistance. In both cases, the virus was able to replicate and move from cell to cell but was unable to reach the phloem. The authors suggest that resistance to viral entrance to the phloem seems to be a general strategy in melon. This opens up new possibilities for using different breeding approaches based on genes, which control different steps of the viral cycle to promote resistances to different viruses.

The cucumber vein yellowing virus (CVYV) causes severe yield losses in cucurbit crops across Mediterranean countries. Pujol et al. identified *CsCvy-1*, a monogenic locus with incomplete dominance for CVYV resistance, in a long Dutch cucumber, which can explain more than 80% of the variance. A BSA (bulked segregant analysis)-Seq and an additional linkage analysis placed *CsCvy-1* in a 625 kb region of cucumber Chromosome 5 (Chr5). There are 24 annotated genes in this region including two *RNA-dependent RNA polymerase* (*RDR1a* and *RDR1b*) genes as potential candidates for *CsCvy-1*. RDRs are critical players in RNA silencing pathways involved in the process of amplification of double-stranded RNAs that activate gene silencing after nuclease processing.

Sáez et al. conducted a QTL analysis for resistance to the Tomato leaf curl New Delhi virus (ToLCNDV) in two pumpkin (*Cucurbita moschata*) accessions (PI 419083 and PI 604506). Allelism tests indicated that both lines carried the same monogenic, recessive locus. Consistent with this, QTL mapping identified a major-effect QTL for ToLCNDV resistance on Chr8, which was also confirmed in the interspecific *C. moschata* × *C. pepo* segregating populations, but the resistance level seemed to be affected by the *C. pepo* genetic background. A comparative analysis indicated that this major-effect QTL was located in a syntenic region on melon Chr11 which was previously shown to harbor a locus for ToLCNDV resistance.

Two other articles reported microRNA (miRNA)-regulated resistance to target leaf spot (TLS, causal agent *Corynespora cassiicola*) and QTL mapping of Fusarium wilt (FW, caused by *Fusarium oxysporum* f. sp. *cucumerinum*) resistances in cucumber. Wang X. et al. studied the association of four miRNAs, miR164d, miR396b, Novel-miR1, and Novel-miR7, with TLS resistance. The four miRNAs inhibit their target genes *NAC transcription factor, APE* (for anthranilate phosphoribosyltransferase), *4CL* (coding for 4-coumarate: CoA ligase), and *PAL* (coding for phenylalanine ammonia-lyase), respectively. Transient expression of the four miRNAs and the corresponding four target genes in cucumber cotyledons, resulted in reduced and enhanced TLS resistance, respectively. Overexpression of *4CL, PAL*, and *novel-miRNA1/7* down-regulated lignin synthesis suggesting that the two miRNAs are associated with TLS resistance through regulating flavonoid biosynthesis. In another article, Dong J. et al. conducted QTL mapping of FW resistance in the cucumber line “Rijiecheng.” A major-effect QTL, *fw2.1* was detected in a 1.91-Mb-long region of cucumber Chr2. Further linkage analysis narrowed the QTL into a 600 kb region containing ~80 annotated genes. RNA-Seq revealed five genes in this region which were up-regulated in the FW resistant parental line. This QTL was adjacent to two FW resistance QTL previously detected in this region.

## QTL Mapping for Abiotic Stress Tolerances in Cucumber and Melon

Two articles in this Research Topic report on QTL mapping of abiotic stress tolerances. Dong S. et al. conducted QTL mapping for low temperature tolerance (LTT) in cucumber seedlings. Phenotyping was performed in replicated trials using an F_2:3_ population and a low-temperature injury index (LTII) that was of quantitative inheritance. Three QTL, *qLTT5.1, qLTT6.1*, and *qLTT6.2* were detected, with the major-effect QTL *qLTT6.2* accounting for ~25% of the observed phenotypic variance. *In silico* BSA and qPCR (quantitative real-time PCR) in a re-sequenced cucumber core collection identified a 42-kb region of the *qLTT6.2* locus with *Csa6G445210* (coding for an auxin response factor), and *Csa6G445230* (coding for an ethylene-responsive transmembrane protein) being the most possible candidates for this QTL.

In another study in melon, Branham et al. report QTL mapping for tolerance to sulfur phytotoxicity and the development of markers useful for melon breeding programs. Sulfur is widely used in cucurbit cultivation to control powdery mildew, although it is known that several accessions can develop a phytotoxicity reaction leading to plant death. The use of the MR-1 × AY RIL (recombinant inbred line) population that segregates for sulfur tolerance allowed the identification of one major (*qSulf-1* on Chr1) and two minor (*qSulf-8* on Chr8, and *qSulf-12* on Chr12) QTL. The authors also provided a set of KASP (Competitive allele specific PCR) markers tightly linked to *qSulf-1*, which can immediately be incorporated into melon breeding programs.

## Molecular Mapping of Fruit Yield and Quality-Related Traits

For all cucurbits, fruit is the main target for breeding improvement. Important attributes affecting cucurbit fruit yield, and quality may include fruit size/shape, fruit external (rind/skin color, stripe pattern, fruit spines, warts, and texture), and internal (flavor/taste, nutritional content, flesh color) qualities. In this Research Topic, seven articles described studies on fruit quality-related traits in cucumber, watermelon and bottle gourd. Lycopene content and flesh color are important fruit nutritional and appearance attributes for watermelon. Wang C. et al. conducted linkage analysis in a red × pale yellow flesh F_2_ population and found that the red flesh color was controlled by a recessive gene, which was located in a 392 kb region on Chr4. Further linkage and association analyses narrowed the candidate gene down to a ~41 kb region. Additional evidence supported the *lycopene* β*-cyclase* (*LCYB, Cla005011*) gene as the candidate that regulates flesh color changes at the protein level. Cleaved amplified polymorphic sequence (CAPS) markers were developed to differentiate red vs. yellow flesh colors in watermelon genotypes that could be used in marker-assisted breeding.

Bitter fruit is undesirable for consumption in bottle gourd (*Lagenaria siceraria*). Wu et al. phenotyped an F_2:3_ population with a trained panel of sensory analysis and found two complementary genes underlying fruit bitterness in bottle gourd, which was consistent with the QTL mapping result in this population. The two QTL, *QBt.1* and *QBt.2*, were located in a region of 1.6-Mbp and 1.9-Mbp on Chr6, and Chr7, respectively, which were validated in an advanced bulked segregant analysis (A-BSA). Sequence-based comparative analysis suggested that the causal genes underlying *QBt.1* and *QBt.2* may not be direct orthologs of the reported bitterness genes in cucumber, melon and watermelon.

Among the four articles on cucurbit external fruit quality attributes, Liu et al. investigated the inheritance of rind (skin) colors in watermelon. BSA-Seq and fine mapping with a yellow × green F_2_ population mapped the *yellow rind* (*Clyr*) locus in a 91.42 kb region on Chr4. The authors also conducted comparative transcriptomic analysis in the two parental lines at different fruit development stages and identified genes and pathways related to rind pigment metabolism. Rett-Cadman et al. studied developmental changes and natural variation of cucumber fruit surface properties. They first conducted a microscopic investigation of the fruit surface in two cucumber lines (CL9930 and Gy14) at different development stages and found significant differences in cuticle thickness, depth of wax intercalation between epidermal cells, epidermal cell size and shape, and number and size of lipid droplets in the two lines. QTL mapping in the Gy14 × CL9930 RIL population revealed strong QTL for epidermal cell height, cuticle thickness, intercalation depth, and diameter of lipid droplets co-localized on cucumber Chr1. Fine mapping combined with gene expression profiling suggested a small number of candidate genes underlying these traits. Tissue specificity, developmental analysis of expression, allelic diversity, and gene function analyses identified the transcription factor *CsSHINE1/CsWIN1* (*CsV3_1g030200*) as a source of natural variation for cucumber fruit epidermal traits. Pericarp wax on cucumber fruit skin may affect consumer preference and marketability. Grafting of cucumber onto pumpkin rootstock (*Cucurbita moschata*) often produces glossy fruits. Zhang J. et al. conducted transcriptome profiling, genome-wide DNA methylation sequencing, and wax metabolite analysis in the pericarps of self-rooted vs. grafted cucumbers, and found that the AP2/ERF-type transcription factor gene *CsWIN1* (*Csa3G878210*) was methylated and up-regulated in grafted compared to self-rooted scions. The increased expression of *CsWIN1* was positively correlated with expression of several key wax biosynthesis genes and the accumulation of wax esters in grafted cucumber pericarp. The glossier appearance of grafted pericarp seemed to be the result of higher wax ester content and higher integration of small trichomes in the pericarp. It is interesting to see that the two different members of the AP2/ERF-type transcription factor gene family control different aspects of fruit epidermal features.

The fruit spine is also an important external fruit quality trait. The MYB transcription factor gene *CsTRY* (*Csa5G139610*) is a negative regulator for fruit spine initiation. Zhang L. et al. found that in plants overexpressing *CsTRY*, the expression of key genes in the flavonoid synthesis pathway was repressed, suggesting that CsTRY not only regulates the development of fruit spines, but also functions in the synthesis of flavonoids, acting as the repressor of anthocyanin synthesis.

Cucumber is consumed when still immature. Over ripe fruit affects its market value. Cheng et al. isolated and characterized a MADS-box gene, *Cucumis sativus SHATTERPROOF* (*CsSHP*) in cucumber that may participate in fruit maturation through the ABA pathway and floral organ specification *via* interaction with CsSEPs (SEPALLATA).

## QTL for Seed-Related Traits

In many countries, seed are also the end products for watermelon and pumpkin/squash (*Cucurbita* spp.) production. There is considerable variation in seed color, size, and shape in these crops. Four articles reported on a genetic analysis and development of molecular markers for seed color and size in watermelon and pumpkin, and a comparative analysis QTL for seed size variation among cucurbits. Paudel et al. examined the previously established four-gene model (*R, T1, W*, and *D*) explaining seed coat color variation in watermelon. QTL-seq and genotyping-by-sequencing (GBS) analyses mapped *R, T1, W*, and *D* on Chr3, 5, 6, and 8, respectively. They also developed KASP assays and SNP markers useful for incorporating each of these gene loci in watermelon breeding programs. In another study, Li B. et al. conducted a genetic mapping analysis and identified a *polyphenol oxidase* (*PPO*) gene (*Cla019481*) as the candidate for the *ClCS1* locus controlling the black seed color in watermelon. The black color was proposed to be due to a frameshift mutation in this *PPO* gene that is involved in melanin oxidation during melanin biosynthesis.

In pumpkin (*C. maxima*), based on a high-density SNP-based genetic map, Wang Y. et al. identified 10 QTL for seed width (SW), seed length (SL), and hundred-seed weight (HSW). Based on gene annotation and non-synonymous SNPs in the major SL and SW-associated regions, two genes coding for a VQ motif and an E3 ubiquitin-protein ligase were proposed to be the potential candidates influencing SL, while an F-box and leucine-rich repeat (LRR) domain-containing protein is the potential regulator for SW in *C. maxima*.

Guo et al. conducted a literature review on seed size (SS) QTL identified in watermelon, pumpkin/squash, cucumber, and melon, and inferred consensus SS QTL based on their physical positions in respective draft genomes. Among them, four from watermelon (*ClSS2.2, ClSS6.1, ClSS6.2*, and *ClSS8.2*), two from cucumber (*CsSS4.1* and *CsSS5.1*), and one from melon (*CmSS11.1*) were a major-effect, stable QTL for seed size and weight, which were located in syntenic regions across different genomes, suggesting possible structural, and functional conservation of some important genes for seed size control in cucurbit crops.

## Plant Architecture in Watermelon and Sex Expression in Cucurbits

Plant architecture is important for crop improvement. There is great interest by breeders and growers in the development of dwarf watermelon varieties useful for high plant density cultivation and high yield production. Zhu et al. conducted BSA-Seq of F_2_ plants derived from a cross between a normal-height and dwarf watermelon inbred lines. They identified a gene (*Cla010337*) coding for an ATP-binding cassette transporter (ABC transporter) protein with two SNPs associated with the dwarfness. A derived CAPS (dCAPS) marker that co-segregates with the dwarf trait was validated using the F_2_ population and a germplasm collection of 165 watermelon accessions. This dCAPS marker can be used in a marker-assisted selection (MAS) for the development of dwarf watermelon cultivars.

In cucurbits, sex determination is critically associated with fruit earliness, yield, and quality. Li D et al. reviewed this important topic on how sex determination genes and their interactions determine sex in cucurbit crops. Six sex morphs could be found based on the distribution of female, male, or bisexual flowers on a plant. So far, five orthologous genes involved in sex determination have been cloned, and their various combinations and expression patterns can explain all the identified sex types in cucurbits. The phytohormone ethylene plays a critical role in sex expression in cucurbits. Two ethylene signaling components, CsERF110 and CsERF31, have recently been identified, which may help in exploring the ethylene signaling-mediated interactions among sex-related genes. The authors proposed a nomenclature to name genes and sex morphs across different cucurbit crops, which is at present rather confusing.

## Conclusions

This Research Topic provides readers with updates of translational research in cucurbit crops, many updates of which are not only of importance for cucurbit breeding, but also elucidate basic concepts of cucurbit development and response to biotic and abiotic stressors. The editors thank all contributors to this Research Topic.

## Author Contributions

All authors listed have made a substantial, direct and intellectual contribution to the work, and approved it for publication.

## Conflict of Interest

The authors declare that the research was conducted in the absence of any commercial or financial relationships that could be construed as a potential conflict of interest.
